# Supplementary material for: A Likelihood Approach for Real-Time Calibration of Stochastic Compartmental Epidemic Models
Source: PLoS Comput Biol. 2017 Jan 17;13(1):e1005257. doi: 10.1371/journal.pcbi.1005257 (PMC5240920; doi:10.1371/journal.pcbi.1005257)
Supplement: S1 File — (TAR.GZ) [file pcbi.1005257.s014.tar.gz › HSPH_Online-SI-Revision/Additions/MSS11/n100-extreme-SEITR/n100-extreme-SEITR_NEW_table-4.pdf]

1	R0	{1.38654, 9.85565, 1.}	{1.45253, 4.01667, 0.}	{{1.44762, 4.00571, 0.}, {1.47488, 4.06661, 0.}}	{4.75913, 59.245, 100.}	inf	7628.	3033.91	{2797.8, 3259.96}	60.2266	0.401225	newly	7106.	6620.63	{6122., 7049.}	7.44258	0.0340643	Reff	1.09645	1.28714	{1.2808, 1.30524}	17.3917	ar	92379.	55075.3	{53975., 56427.}	40.3811
2	R0	{2.36629, 12.2029, 0.1}	{2.77252, 9.32887, 0.13}	{{2.17292, 6.16453, 0.}, {2.99092, 10.3743, 0.}}	{20.6989, 23.5521, 212.}	inf	2781.	2239.14	{1477.8, 2605.26}	19.5009	0.105342	newly	3034.	3261.31	{3007., 3543.}	8.78148	0.0362947	Reff	2.22479	2.67289	{2.11198, 2.8766}	22.5421	ar	99022.	91459.6	{83853., 94066.}	7.63706
3	R0	{2.32253, 12.836, 1.}	{1.68185, 3.74753, 0.41}	{{1.39162, 1.89883, 0.}, {1.9862, 6.28417, 1.}}	{27.5856, 70.8046, 67.}	inf	1753.	777.203	{454.038, 1204.45}	70.527	0.56066	newly	2684.	2837.85	{2022., 3624.}	20.1949	0.0834272	Reff	2.19238	1.64786	{1.37161, 1.93816}	24.8367	ar	98997.	66419.3	{51069., 79210.}	32.9078
4	R0	{1.79075, 11.2058, 1.}	{1.6489, 4.75244, 0.27}	{{1.53501, 4.02511, 0.}, {1.81768, 5.68021, 1.}}	{11.135, 57.5894, 81.}	inf	2803.	1786.12	{1306.94, 2301.65}	56.0069	0.369979	newly	3933.	4019.85	{3480., 4489.}	7.82075	0.033463	Reff	1.61667	1.57145	{1.46544, 1.72694}	9.01942	ar	96920.	65590.3	{60074., 73986.}	32.3253
5	R0	{2.26952, 9.62371, 0.1}	{1.79416, 4.0145, 0.04}	{{1.65456, 3.37285, 0.}, {1.94705, 4.66877, 0.}}	{21.1064, 58.2853, 132.}	inf	1270.	739.226	{459.816, 1026.24}	62.5519	0.450561	newly	3231.	2618.99	{1999., 3172.}	19.905	0.10342	Reff	2.17415	1.7634	{1.62667, 1.91275}	19.0917	ar	98881.	71940.4	{66415., 78259.}	27.2454
6	R0	{1.57191, 6.89004, 0.1}	{1.65632, 3.39408, 0.04}	{{1.39162, 1.89883, 0.}, {1.88643, 4.56746, 0.}}	{12.1904, 53.719, 132.}	inf	1343.	951.46	{480.221, 1437.14}	54.2012	0.378876	newly	3690.	3894.62	{2754., 4972.}	22.1626	0.0997575	Reff	1.49482	1.61287	{1.35999, 1.83278}	13.1934	ar	95046.	64350.8	{50060., 76202.}	32.2951
7	R0	{1.99226, 17.2339, 1.}	{1.45877, 4.12361, 0.18}	{{1.44762, 4.00571, 0.}, {1.44762, 4.00571, 0.}}	{26.778, 76.0727, 106.}	inf	7484.	3280.01	{2983.32, 3518.14}	75.3753	0.611693	newly	6848.	6864.56	{6369., 7337.}	4.47956	0.0194039	Reff	1.39466	1.27663	{1.26506, 1.27308}	8.84682	ar	97992.	55264.5	{53818., 55735.}	43.603
8	R0	{2.63369, 15.1879, 0.1}	{2.52594, 8.93458, 0.07}	{{1.39162, 1.89883, 0.}, {2.80387, 10.6536, 0.}}	{10.2053, 41.173, 158.}	inf	3695.	3394.51	{1252.23, 4092.33}	43.1596	0.281175	newly	4729.	4831.5	{4298., 5136.}	9.30556	0.0362555	Reff	2.31741	2.37665	{1.34933, 2.63169}	14.0108	ar	99451.	86149.2	{50942., 92614.}	13.3752
9	R0	{2.53271, 9.90552, 0.1}	{2.68922, 7.82145, 0.15}	{{2.45236, 6.2492, 0.}, {2.77394, 8.24535, 1.}}	{10.5804, 21.0395, 226.}	inf	823.	858.303	{701.106, 1013.1}	31.9887	0.174713	newly	2227.	1738.33	{1462., 2009.}	22.9201	0.115534	Reff	2.46668	2.65182	{2.42079, 2.73905}	11.4832	ar	99355.	90783.6	{88807., 92463.}	8.62705
10	R0	{2.48791, 18.9003, 2.}	{2.29438, 10.405, 1.02}	{{1.74092, 5.83416, 0.}, {2.5898, 12.9574, 2.}}	{9.55341, 44.9481, 49.}	inf	5017.	4466.68	{2457.17, 5628.42}	46.8822	0.290602	newly	4646.	4362.68	{4131., 4558.}	6.45932	0.0292282	Reff	2.05964	2.10184	{1.6322, 2.34575}	10.7432	ar	99291.	84699.2	{70800., 90313.}	14.696
11	R0	{2.84414, 18.4765, 1.}	{1.77259, 5.40204, 0.62}	{{1.53501, 4.02511, 0.}, {1.81768, 5.68021, 1.}}	{37.6757, 70.7626, 40.}	inf	3971.	1809.86	{1340.32, 2136.66}	71.8528	0.557102	newly	4035.	3710.46	{3267., 4069.}	9.09343	0.0426798	Reff	2.47881	1.69685	{1.47692, 1.74603}	31.5456	ar	99654.	71513.3	{60279., 74818.}	28.2384
12	R0	{2.29369, 14.622, 0.1}	{2.16315, 7.50221, 0.18}	{{1.98388, 6.25659, 0.}, {2.26582, 8.18891, 0.}}	{6.07276, 48.6923, 260.}	inf	4819.	4182.3	{3375.53, 4742.39}	47.0595	0.280371	newly	5911.	6174.46	{5774., 6493.}	5.57097	0.0234309	Reff	1.91186	1.98693	{1.83545, 2.08007}	6.86584	ar	98982.	83278.4	{79088., 85931.}	15.8651
13	R0	{2.73413, 16.7529, 1.}	{2.39945, 8.82604, 0.45}	{{1.98388, 6.25659, 0.}, {2.9884, 12.8326, 1.}}	{15.6215, 47.3164, 73.}	inf	4263.	3580.33	{2437.34, 4939.32}	47.0365	0.291901	newly	4789.	4732.11	{4334., 5086.}	4.96701	0.0218399	Reff	2.35852	2.24482	{1.87748, 2.75895}	12.5466	ar	99543.	86210.6	{79116., 93889.}	13.3936
14	R0	{1.79554, 16.5943, 2.}	{1.66561, 7.19473, 0.99}	{{1.67405, 7.28757, 1.}, {1.67405, 7.28757, 1.}}	{7.23637, 56.6433, 50.5}	inf	6015.	4140.35	{3991.05, 4356.95}	58.9455	0.390212	newly	5421.	5030.01	{4783., 5256.}	7.24719	0.0329162	Reff	1.3756	1.47503	{1.47852, 1.48485}	7.91919	ar	96821.	67340.	{67379., 68459.}	30.449
15	R0	{1.91429, 13.7771, 1.}	{1.4921, 4.11781, 0.06}	{{1.47488, 3.83841, 0.}, {1.47608, 4.06661, 0.}}	{22.0549, 70.1112, 100.}	inf	5411.	2489.86	{2167.52, 2733.59}	71.3381	0.547265	newly	6031.	5690.2	{5113., 6211.}	7.58349	0.0346104	Reff	1.53354	1.36829	{1.3495, 1.36043}	11.2517	ar	97541.	57600.1	{56042., 57703.}	40.9478
16	R0	{1.42094, 10.5871, 2.}	{1.83784, 8.07696, 1.01}	{{1.24751, 2.5364, 0.}, {2.36011, 13.1698, 2.}}	{36.2596, 47.6166, 50.5}	inf	3733.	4235.37	{1232.62, 6948.95}	46.2944	0.315028	newly	4920.	4772.15	{4094., 5261.}	7.04329	0.0326513	Reff	1.20628	1.6408	{1.15693, 2.06482}	38.2126	ar	93044.	65814.	{36852., 87350.}	29.2657
17	R0	{1.45515, 6.16466, 0.1}	{1.97161, 5.14308, 0.17}	{{1.65456, 3.37285, 0.}, {2.17292, 6.48739, 1.}}	{35.6217, 20.7997, 242.}	inf	733.	840.083	{471.049, 1131.42}	23.8666	0.127662	newly	2188.	2290.64	{1719., 2863.}	15.8419	0.0672322	Reff	1.41733	1.93687	{1.62915, 2.13357}	36.7186	ar	93254.	77192.7	{66741., 84077.}	17.237
18	R0	{2.85669, 13.2416, 0.1}	{2.82811, 9.69566, 0.02}	{{2.17292, 6.16453, 0.}, {2.99092, 10.3908, 0.}}	{6.23699, 26.7788, 116.}	inf	1540.	1510.9	{978.459, 1725.66}	38.8794	0.229916	newly	2807.	2151.52	{1936., 2353.}	23.3516	0.117154	Reff	2.71777	2.76032	{2.13441, 2.91383}	8.29606	ar	99644.	91593.9	{84214., 94009.}	8.07886
19	R0	{2.56417, 17.9558, 0.1}	{1.58802, 4.47038, 0.}	{{1.47608, 3.83841, 0.}, {1.74592, 5.58477, 0.}}	{38.0689, 75.1034, 100.}	inf	4153.	1544.73	{1198.6, 1939.96}	76.9581	0.644918	newly	4054.	3653.74	{3178., 4065.}	10.8949	0.0515563	Reff	2.21442	1.51902	{1.41501, 1.66141}	31.4034	ar	99389.	62949.8	{56615., 71000.}	36.6632
20	R0	{2.27517, 11.1864, 0.1}	{2.87458, 9.87177, 0.08}	{{2.27423, 6.876, 0.}, {2.99092, 10.3908, 0.}}	{27.374, 11.7521, 170.}	inf	2051.	2720.56	{1984.9, 3061.06}	11.7425	0.0614641	newly	3692.	3693.75	{3432., 4006.}	4.98185	0.0219345	Reff	2.12451	2.75237	{2.20649, 2.86223}	30.0086	ar	98894.	92636.2	{86010., 94068.}	6.32778
21	R0	{2.35735, 10.4735, 0.1}	{2.6646, 7.34116, 0.47}	{{2.49133, 6.22691, 0.}, {2.9751, 8.95585, 1.}}	{15.5878, 29.9073, 478.}	inf	1087.	1281.84	{994.021, 1557.04}	20.8306	0.11976	newly	2417.	2838.85	{2446., 3335.}	19.7935	0.0777697	Reff	2.2751	2.60868	{2.43869, 2.90662}	17.03	ar	99036.	90101.4	{88996., 93903.}	9.02159
22	R0	{1.79551, 11.9179, 2.}	{2.02913, 7.80826, 0.08}	{{1.737, 5.43578, 0.}, {2.22376, 9.35432, 0.}}	{17.1347, 35.1961, 97.}	inf	4439.	4485.46	{3100.18, 5584.72}	36.0989	0.212165	newly	5958.	5855.14	{5521., 6146.}	3.59953	0.0158638	Reff	1.5037	1.83639	{1.59629, 2.00261}	22.8795	ar	97033.	78593.	{70375., 85164.}	19.0038
23	R0	{1.23545, 6.31687, 0.1}	{1.61804, 4.6322, 0.02}	{{1.53501, 4.02511, 0.}, {1.74592, 5.58477, 0.}}	{30.9677, 27.8734, 118.}	inf	2814.	2781.68	{2186.69, 3469.89}	31.5956	0.170334	newly	6598.	6013.02	{5509., 6503.}	9.15277	0.0426827	Reff	1.0892	1.49122	{1.41728, 1.6012}	36.91	ar	88739.	64439.9	{59878., 70919.}	27.3827
24	R0	{2.24089, 10.1789, 0.1}	{2.53126, 7.14168, 0.11}	{{1.94705, 4.66877, 0.}, {2.77394, 8.24535, 0.}}	{21.6012, 29.8768, 192.}	inf	976.	968.788	{509.936, 1241.83}	30.8047	0.181037	newly	2027.	2114.9	{1698., 2484.}	12.2003	0.051415	Reff	2.17337	2.48952	{1.91908, 2.73039}	22.4966	ar	98868.	87454.4	{77750., 92432.}	11.5443
25	R0	{1.57298, 10.2607, 0.1}	{2.49497, 12.1791, 0.}	{{2.52368, 12.2207, 0.}, {2.52588, 12.5037, 0.}}	{58.6142, 21.2855, 100.}	inf	2641.	4734.21	{4526.25, 5063.93}	22.2972	0.0892119	newly	3941.	3957.39	{3694., 4234.}	4.14539	0.0178476	Reff	1.41523	2.29044	{2.30818, 2.31985}	61.8428	ar	94957.	88922.8	{89258., 89770.}	6.35472
26	R0	{1.2154, 8.2576, 0.1}	{1.46808, 4.21572, 0.15}	{{1.44762, 4.00571, 0.}, {1.44762, 4.00571, 0.}}	{20.79, 49.0457, 236.}	inf	4010.	3079.72	{2725.36, 3288.55}	48.0449	0.289576	newly	6408.	6412.13	{5983., 6836.}	4.29448	0.0186387	Reff	0.993966	1.30021	{1.28171, 1.28886}	30.8103	ar	88421.	55588.	{53620., 55953.}	37.1326
27	R0	{2.22841, 18.7983, 2.}	{1.9781, 8.96437, 0.}	{{1.93419, 8.56054, 0.}, {2.27199, 11.6669, 0.}}	{11.741, 52.3129, 100.}	inf	7582.	6456.05	{6028.45, 8076.41}	52.301	0.323673	newly	6511.	6517.97	{6292., 6695.}	2.05606	0.00893258	Reff	1.58429	1.69557	{1.66067, 1.90608}	7.0244	ar	98756.	78755.9	{77376., 85528.}	20.2521
28	R0	{1.27778, 5.36767, 1.}	{1.42362, 2.19033, 0.25}	{{1.39162, 1.89883, 0.}, {1.43965, 2.60601, 1.}}	{11.4134, 61.9051, 79.}	inf	966.	514.318	{185.91, 738.567}	61.615	0.461338	newly	2971.	3290.61	{1322., 5155.}	39.3554	0.181431	Reff	1.23118	1.39556	{1.36204, 1.41218}	13.3517	ar	89843.	51895.1	{49500., 54054.}	42.3336
29	R0	{2.38042, 15.8901, 2.}	{2.26844, 9.27263, 0.36}	{{2.12684, 8.23415, 0.}, {2.68687, 12.2271, 1.}}	{11.8121, 41.6452, 82.}	inf	3337.	3117.94	{2542.25, 4146.72}	40.5654	0.236281	newly	3642.	3655.87	{3475., 3840.}	3.28199	0.0142473	Reff	2.11469	2.13732	{2.01149, 2.50586}	9.56824	ar	99112.	84667.5	{82510., 91375.}	14.5739
30	R0	{2.53188, 12.7607, 0.1}	{2.5964, 7.47647, 0.12}	{{1.94705, 4.66877, 0.}, {2.77394, 8.24535, 0.}}	{13.1787, 41.4102, 202.}	inf	1016.	956.834	{556.055, 1160.54}	32.1875	0.186517	newly	1649.	1989.02	{1669., 2301.}	22.6525	0.0872068	Reff	2.45506	2.55501	{1.92165, 2.73196}	14.0973	ar	99317.	88862.3	{77926., 92408.}	10.5266
31	R0	{1.24482, 10.5471, 1.}	{1.30579, 3.27896, 0.14}	{{1.24751, 2.5364, 0.}, {1.24751, 2.5364, 0.}}	{4.89786, 72.3588, 100.}	inf	5483.	2601.78	{1740.21, 2460.21}	72.5853	0.591249	newly	6713.	5921.59	{5053., 6607.}	12.6264	0.0605205	Reff	0.914831	1.10393	{1.06322, 1.07224}	20.6704	ar	89097.	40324.4	{35820., 39282.}	54.741
32	R0	{2.30446, 17.9607, 1.}	{2.71473, 13.8396, 1.96}	{{2.7346, 14.003, 2.}, {2.7346, 14.003, 2.}}	{18.7814, 22.9451, 100.}	inf	5816.	7785.54	{7682.93, 8042.07}	20.8384	0.103701	newly	5244.	5514.43	{5360., 5660.}	5.15694	0.0217373	Reff	1.82382	2.3612	{2.37166, 2.38188}	29.9797	ar	99028.	91460.1	{91665., 92041.}	7.64216
33	R0	{2.31579, 11.8236, 2.}	{1.65157, 3.57642, 0.61}	{{1.43965, 2.60601, 0.}, {1.88643, 4.61852, 1.}}	{28.6823, 69.7519, 69.5}	inf	3092.	1371.3	{839.089, 1985.04}	70.9039	0.56391	newly	5187.	4877.5	{3988., 5811.}	12.3038	0.0572699	Reff	2.08296	1.59085	{1.38934, 1.80865}	23.6257	ar	99012.	65117.3	{53400., 76165.}	34.2329
34	R0	{1.6678, 10.6744, 1.}	{1.5778, 4.34693, 0.}	{{1.53501, 4.02511, 0.}, {1.74592, 5.58477, 0.}}	{7.32527, 59.2771, 100.}	inf	4713.	2981.12	{2479.14, 3844.26}	58.1656	0.383971	newly	6918.	6703.87	{6052., 7259.}	5.69254	0.025599	Reff	1.37288	1.43817	{1.39876, 1.5766}	4.81509	ar	95809.	62676.1	{59786., 71092.}	34.5822
35	R0	{1.65476, 7.07559, 0.1}	{2.11254, 5.68958, 0.33}	{{1.88643, 4.56746, 0.}, {2.45236, 7.26016, 1.}}	{27.6659, 23.5226, 382.}	inf	893.	1022.28	{690.78, 1442.45}	25.067	0.131931	newly	2514.	2563.09	{2162., 2934.}	10.294	0.0446229	Reff	1.60409	2.07093	{1.84906, 2.39877}	29.103	ar	95746.	81190.9	{75349., 88753.}	15.2018
36	R0	{2.71807, 18.5959, 0.1}	{2.11252, 7.61597, 0.59}	{{1.81768, 5.68021, 0.}, {2.2983, 8.81055, 1.}}	{22.2787, 59.0449, 578.}	inf	3397.	2113.98	{1540.32, 2557.7}	60.1136	0.407076	newly	3163.	3168.25	{2905., 3394.}	5.38508	0.0233319	Reff	2.4286	2.02588	{1.75263, 2.19765}	16.6532	ar	99540.	81742.7	{73781., 86301.}	17.8795
37	R0	{2.6202, 12.7552, 0.1}	{1.91671, 4.61605, 0.04}	{{1.82289, 4.40955, 0.}, {1.94705, 4.66877, 0.}}	{26.8487, 63.8104, 132.}	inf	1248.	691.309	{471.946, 821.124}	62.2854	0.432821	newly	2178.	2197.22	{1585., 2594.}	13.977	0.0621844	Reff	2.51914	1.88851	{1.79699, 1.92186}	25.0336	ar	99426.	76974.5	{74117., 78512.}	22.5811
38	R0	{1.47672, 9.81651, 1.}	{2.49394, 13.0657, 1.04}	{{2.14754, 9.98575, 1.}, {2.5684, 13.7324, 1.}}	{68.8835, 33.72, 4.}	inf	4210.	7953.8	{6005.91, 8538.39}	25.834	0.0991858	newly	6266.	5739.05	{5582., 5892.}	8.40967	0.0382422	Reff	1.23	2.14332	{1.88867, 2.20283}	74.2532	ar	93815.	88893.7	{83318., 90231.}	5.24571
39	R0	{1.90004, 12.3313, 1.}	{1.70488, 5.46147, 0.11}	{{1.47608, 3.83841, 0.}, {1.75359, 5.92488, 1.}}	{12.5146, 55.8732, 89.}	inf	3615.	2493.98	{1749.51, 2861.25}	55.4305	0.362077	newly	4980.	4686.08	{4231., 5081.}	7.33494	0.0336096	Reff	1.65284	1.59611	{1.39608, 1.63967}	6.32442	ar	97413.	68234.	{57500., 71564.}	29.9539
40	R0	{2.64218, 18.5237, 2.}	{1.47991, 3.13233, 1.39}	{{1.43965, 2.60601, 0.}, {1.53501, 4.02511, 2.}}	{43.9891, 83.0901, 31.5}	inf	6412.	1944.7	{1466.54, 2390.41}	82.1783	0.756923	newly	6088.	6587.31	{5272., 7802.}	14.2571	0.0578221	Reff	2.0572	1.37974	{1.3419, 1.42717}	32.931	ar	99474.	56756.	{53746., 60664.}	42.9439
41	R0	{1.6945, 7.17996, 0.1}	{2.72191, 9.25906, 0.09}	{{2.02938, 5.7296, 0.}, {2.93906, 10.5991, 0.}}	{60.6789, 38.5561, 174.}	inf	498.	783.845	{516.806, 956.658}	29.4911	0.116392	newly	1333.	1203.16	{979., 1377.}	13.2528	0.0627838	Reff	1.66739	2.68777	{2.0122, 2.90177}	61.2291	ar	96332.	90531.2	{80744., 93727.}	6.0217
42	R0	{1.84559, 18.0074, 1.}	{1.87627, 9.51971, 0.79}	{{1.24751, 2.5364, 0.}, {2.1245, 12.2645, 1.}}	{19.2181, 47.1345, 31.}	inf	7319.	6840.11	{1697.54, 8989.3}	47.7136	0.362599	newly	6151.	5946.58	{5197., 6256.}	4.26662	0.0201955	Reff	1.29117	1.55526	{1.10334, 1.73458}	27.943	ar	97358.	70268.1	{36911., 82992.}	27.8251
43	R0	{2.24965, 17.0361, 2.}	{2.83407, 15.3393, 1.96}	{{2.86118, 15.5722, 2.}, {2.86118, 15.5722, 2.}}	{27.3014, 9.95976, 2.}	inf	4414.	6263.62	{6168.45, 6517.13}	12.3724	0.0624469	newly	4281.	4078.03	{3945., 4204.}	4.81733	0.0215632	Reff	1.90728	2.55168	{2.56967, 2.57933}	34.7987	ar	98770.	92327.3	{92803., 93170.}	6.52295
44	R0	{2.54324, 15.7967, 0.1}	{1.9311, 5.90524, 0.14}	{{1.43965, 2.60601, 0.}, {1.9862, 6.28417, 1.}}	{24.0693, 62.6173, 212.}	inf	4842.	2968.58	{1462.19, 3394.18}	61.3516	0.429756	newly	5449.	5722.94	{5300., 6080.}	6.43641	0.0265837	Reff	2.13836	1.80673	{1.37199, 1.86141}	15.5082	ar	99354.	76245.9	{54310., 79677.}	23.2584
45	R0	{2.54943, 14.8032, 0.1}	{2.71563, 10.1507, 0.13}	{{2.59708, 9.39342, 0.}, {2.80387, 10.6536, 1.}}	{6.94415, 31.4287, 204.}	inf	3565.	3904.57	{3522.13, 4298.35}	30.6839	0.160723	newly	4552.	4640.23	{4372., 4902.}	4.00154	0.0171144	Reff	2.25387	2.54424	{2.43533, 2.62626}	13.074	ar	99380.	91609.6	{90204., 92689.}	7.81891
46	R0	{2.37755, 12.6461, 2.}	{1.3921, 1.9059, 0.01}	{{1.39162, 1.89883, 0.}, {1.39162, 1.89883, 0.}}	{41.4479, 84.9289, 99.5}	inf	1629.	386.789	{90.6056, 670.507}	85.043	0.919637	newly	2717.	2923.45	{783., 4979.}	47.3371	0.233884	Reff	2.25026	1.37086	{1.36595, 1.37451}	39.0796	ar	99096.	50430.6	{49330., 51518.}	49.1094
47	R0	{1.69279, 11.6636, 1.}	{1.74199, 5.83245, 0.08}	{{1.74092, 5.83416, 0.}, {1.74092, 5.83416, 0.}}	{2.90653, 49.9945, 92.}	inf	3683.	2753.06	{2571.64, 2951.97}	50.8996	0.309481	newly	4864.	4792.61	{4507., 5149.}	4.15769	0.0183306	Reff	1.4668	1.62331	{1.61884, 1.62819}	10.6698	ar	96214.	70869.9	{70296., 71517.}	26.3414
48	R0	{1.82402, 10.0038, 1.}	{2.43851, 8.93504, 0.21}	{{1.98388, 6.25659, 0.}, {2.74056, 10.6768, 1.}}	{33.6888, 16.2757, 85.}	inf	1751.	2415.64	{1636.34, 3016.01}	18.3766	0.0900435	newly	3183.	3260.95	{2995., 3545.}	5.29783	0.0224801	Reff	1.71321	2.33595	{1.91655, 2.61601}	36.3493	ar	97076.	87268.4	{79141., 92070.}	10.103
49	R0	{1.6424, 11.1667, 2.}	{1.88361, 7.27719, 1.04}	{{1.47608, 3.83841, 0.}, {2.63407, 13.6136, 3.}}	{26.5731, 49.2933, 82.}	inf	5534.	5574.27	{2832.26, 10349.8}	47.6093	0.31767	newly	7611.	7115.59	{6573., 7679.}	7.19419	0.0327651	Reff	1.28365	1.62061	{1.31783, 2.16969}	26.25	ar	95546.	69312.4	{56157., 90925.}	27.4566
50	R0	{2.05638, 14.3048, 1.}	{2.35312, 10.0871, 1.6}	{{1.53501, 4.02511, 0.}, {2.59133, 11.8519, 2.}}	{25.1932, 29.4844, 104.}	inf	4454.	5087.62	{2069.48, 6117.73}	26.5537	0.166187	newly	5024.	5214.63	{5006., 5445.}	4.34335	0.0183185	Reff	1.73898	2.13127	{1.44024, 2.33585}	29.6971	ar	98194.	83904.6	{60881., 90537.}	14.5522
